# Molecular identification and biological activity of a red sea bacterial isolate

**DOI:** 10.1186/s12866-026-04903-1

**Published:** 2026-03-25

**Authors:** Mohamed M. Matar, Yomna N. Elkholy, Walaa A. Negm, Hossam H. Elfeky, Mahmoud A. Yassien

**Affiliations:** 1 Department of Microbiology and Immunology, Faculty of Pharmacy, Alsalam University, Kafr Al Zayat, AlGharbia, 31611 Egypt; 2 Department of Microbiology & Immunology, Faculty of Pharmacy, Organization of African Unity St. Abbassia, Ain Shams University, Cairo, 11566 Egypt; 3 Department of Pharmacognosy, Faculty of Pharmacy, Tanta University, Tanta, 31527 Egypt; 4 Department of Microbiology and Immunology, College of Pharmacy, Suez Canal University, Ismailia, Egypt

**Keywords:** Marine, *Phallusia nigra*, *Heyndrickxia ginsengihumi*, Antimicrobial, Anticancer, Anti-inflammatory, Antioxidant

## Abstract

Marine microorganisms residing in the Red Sea have been recognized as valuable sources of novel natural products with potential medical applications. One distinct bacterial isolate coded MMM3 was isolated from the marine tunicate *Phallusia nigra*, gathered from the Egyptian coast of the Red Sea. The present study aimed to identify and assess the isolated strain’s biological activities. After genomic extraction and PCR amplification, the bacterial strain was identified through 16 S rRNA gene sequencing as *Heyndrickxia ginsengihumi* and deposited in (NCBI) GenBank database under the accession code PX237387. The tested isolate was cultivated in R2A liquid medium for ten days at 25 °C in a shaking incubator set at 220 rpm. The crude extract exhibited effective COX-2 inhibition and antioxidant activity with IC₅₀ value of 107.07 and 451.75 µg/ml, respectively. The crude extract exerted antifungal effect (inhibition zone of 9 mm) against *Candida albicans* ATCC 10,231. In addition, the crude extract demonstrated significant cytotoxic activities against HepG2, A-549, MCF-7 and HCT-116 cell lines with IC_50_ values of 54.67 ± 1.05, 60.19 ± 1.23, 108.79 ± 2.08, and 73.57 ± 1.48 µg/mL, respectively. Regarding the chemical characterization of the crude extract, LC-MS analysis indicated diverse secondary metabolites including linoleic acid, myristoyl glucose-3-phosphate, and 2-acetoxy-4-pentadecylbenzoic acid. The identified metabolites suggest a synergistic contribution to the biological activities of the isolated strain, highlighting its potential as a source of bioactive compounds. The results indicated that the bacteria isolated from Red Sea coast is a promising source of bioactive molecules with medical applications.

## Background

Cancer and microbial infections, standing as the two most significant global health challenges, are affecting people worldwide. Every year, cancer takes up the lives of approximately 10 million people. On the other hand, the perpetual emergence of more drug-resistant pathogens has drastically reduced the efficiency of traditional antimicrobial treatments [1]. Additionally, it is alarming that not only the efficiency of existing drugs is in danger, but also the healthcare expenses and the mortality rates will increase [2]. The mounting pressure caused by resistance and cancer recurrence requires almost immediate action for the creation of more efficacious and safe therapeutic candidates. Over the centuries, natural products, particularly those from microbes, have been the most significant sources in the process of drug discovery [3]. At the same time, the identification of new bioactive compounds from conventional terrestrial sources has reached a standstill, forcing scientists to seek them elsewhere, even in underexplored environments [4].

The Red Sea is a representative example of an environment with extreme conditions - namely the high salinity, the elevated temperatures, and the low nutrient availability and it is this environment that hosts to a myriad of marine organisms that have adapted their metabolisms to survive in such extreme conditions [5]. These significant ecological pressures have been discovered to greatly affect the processing of structurally diverse, highly potent and biologically active secondary metabolites [6]. Marine invertebrates such as tunicates, sponges, and mollusks can cultivate symbiotic or associated microorganisms, that in turn contribute to their defense systems at the chemical levels, which is a widely recognized fact [7]. Among these marine creatures, tunicates have massively gained significant attention due to their capability of hosting various types of microbes that can produce a myriad of pharmacologically active compounds. Tunicates are sessile marine invertebrates belonging to the subphylum Urochordata that are widely distributed in the world’s oceans. They are prolific producers of marine natural products and have been associated with a wide range of biological activities, including antimicrobial, antitumor, and anticancer properties. The symbiotic relationships between tunicates and their associated microbes suggest that many bioactive compounds originate not only from the tunicate host itself but also from its microbial symbionts, which may contribute to defensive metabolites that enhance host survival. These interactions make tunicates and their microbiota a potentially valuable source for the discovery of novel bioactive molecules with ecological.

and biotechnological significance [8] *Phallusia nigra* is a one of marine tunicates that widely distributed in tropical and subtropical coastal waters, including the Red Sea and Indian Ocean. Extracts of *P. nigra* tissues have been reported to contain biologically active compounds with diverse activities. Methanolic extracts of *P. nigra* have shown cytotoxic, antimitotic, and hemolytic effects, suggesting the presence of cytotoxic secondary metabolites in this species. In addition, crude extracts from *P. nigra* exhibited antifouling and antibacterial activities against environmental and laboratory bacterial strains, and significantly reduced settlement of marine fouling organisms in both laboratory and field assays. Chemical analyses of *P. nigra* tissues also revealed multiple metabolites with potential antioxidant and antimicrobial properties. These findings support the potential of *P. nigra* as a promising source of bioactive natural products for marine biotechnology and pharmaceutical research [9]. 

Previous studies have indicated that many bioactive compounds derived from marine associated microorganisms offer multiple benefits [10–12]. These microorganisms, especially those marine Actinomycetota and endophytic bacteria, are the ones that typically possess the biosynthetic gene clusters that encode those activities [13]. Consequently, the reputation of marine-associated bacteria has grown as top-notch producers of natural products capable of healing [14]. The tendency to thrive in extreme marine environments is mainly associated with the capability of the microorganisms to produce unique chemically and biologically potent metabolites [15]. This highlights that the marine microbiome serves as a rich supply of new drugs and biomedical innovations, underscoring its significance in challenging the limits of cancer treatment and antibiotic resistance [16]. The present study aimed to isolate and identify marine-associated bacteria and evaluate its biological activities. In addition, identification of the active metabolites was carried out. The main focus of this work is on *Heyndrickxia ginsengihumi*, a bacterial strain isolated from *Phallusia nigra*, which was selected for its promising preliminary bioactivity. The main achievements of the study include demonstrating a broad spectrum of biological activities including antimicrobial, cytotoxic, anti-inflammatory and antioxidant effects, and providing a detailed metabolite profile of the strain. To the best of our knowledge, this represents the first report of the biological activity and metabolite composition of *H. ginsengihumi* isolated from *P. nigra*, highlighting its novelty and relevance in natural product discovery.

## Materials and methods

### Sample collection and preparation

In August 2024, Three specimens of the tunicate *Phallusia nigra*, were collected as described by Elfeky et al. [17]. from the Red Sea near the coastal waters of Toor Sinai, Egypt (GPS: 28°14’13.3"N 33°36’55.7"E) at water depths ranging from 10 to 12 m. The specimens were identified as *Phallusia nigra*, following standard ascidian taxonomic practice, which is primarily based on external morphological and anatomical characteristics [18]. Identification was conducted by Dr. Hossam H. Elfeky (Department of Microbiology and Immunology, Suez Canal University), who supervised specimen collection. The identification relied on diagnostic features commonly used for species-level recognition of *P. nigra*, including the thick, smooth, jet-black leathery tunic, overall body form, and the relative position and morphology of the oral and atrial siphons, as described in the original species and subsequent biological and taxonomic studies [19]. The specimens were collected by Hossam El-din El-Feky, who holds an Advanced Adventure Diver certification issued by Scuba Diving International (SDI) (Certification No. 1299445).with a license (Cert:# 1299445) who used sterile gloves and immediately placed them into pre-labeled sterile plastic bags filled with seawater at the local temperature toensure the minimum level of stress and contamination. The samples were transported in insulated containers filled with ice and sent to the microbiology laboratory within 7 to 9 h of collection; the microbial viability and community structure were preserved to the maximum extent.

**Fig. 1:**
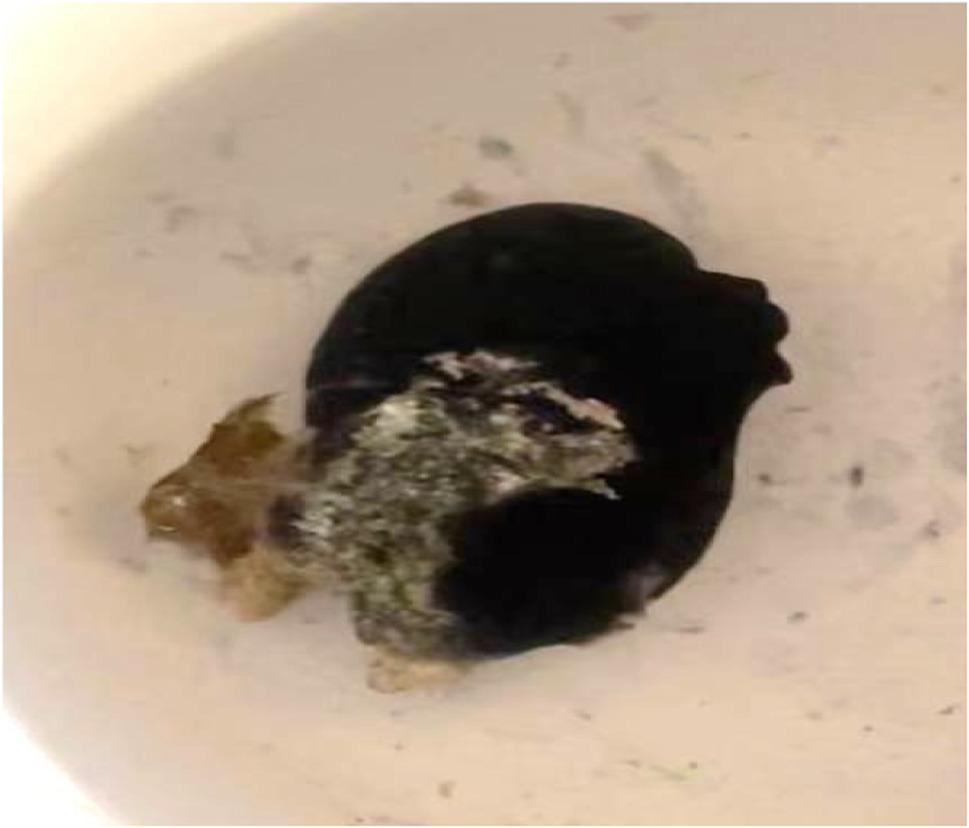
Sample of the tunicate *Phallusia nigra* from the coastal waters of the Red Sea. This specimen served as the source for the isolation of the marine bacterial isolate MMM3

The collected samples were gently washed with sterile natural seawater (NSW) to remove loosely attached epibiotic microorganisms and superficial debris only. A sterile dissection set was used to obtain tissue fragments weighing about 0.5 g. The pieces were cut from the inner tunic surface under a laminar flow hood using aseptic techniques. These tissue samples were placed in sterile Petri dishes to start the extraction of marine-associated organism. A sample of *phallusia nigra* collected from the Red Sea is presented in Fig. 1.

### Isolation of marine-associated microorganism

The isolation of sea-associated bacteria from *Phallusia* nigra was implemented using a culture-based method that allowed both fast-growing and slow-growing microorganisms to be targeted. Interiorly dissected tissue samples were aseptically homogenized in sterile natural seawater, where a sterile glass tissue grinder was employed to release the associated microbial communities into the water. From the homogenate, tenfold serial dilutions (10⁻¹ to 10⁻⁴) were made, and 30 µl from each dilution was distributed onto R2A agar (Casein hydrolysate 0.5 g/L, peptone 0.5 g/L, yeast extract 0.5 g/L, glucose 0.5 g/L, starch 0.5 g/ L, K_2_HPO_4_ 0.3 g/ L, MgSO_4_ 0.024 g/L, sodium pyruvate 0.3 g/L, agar 15 g/L ) supplemented with 2% (w/v) sea salts to create marine osmotic conditions, and the pH was directly adjusted to a final value of 7.2 ± 0.2 before autoclaving for 15 min at 121 °C [20].

The culture medium was pretreated with nystatin (25 µg/mL), cycloheximide (100 µg/mL), and nalidixic acid (25 µg/mL) to improve the selectivity of marine bacteria and inhibit the growth of fungi. After surface inoculation, plates were incubated under aerobic conditions for 14 days at 30 °C [17]. The plates were checked daily for microbial growth. The grown colonies were purified and stored in R2A agar slants at 4 °C, and stocks of glycerol (25% v/v) were prepared and stored for long-term preservation at − 20 °C.

### Preparation of microbial extract

The marine bacterial isolate was inoculated in 300 ml of R2A broth in Erlenmeyer flasks (500-ml) and incubated for 10 days at 25 °C in a shaking incubator at 220 rpm. After incubation, the bacterial cells were filtered from the culture broth using Whatman no.1 filter paper. Extraction of the secondary metabolites from the collected filtrate was carried out using ethyl acetate (equal volume; 1: 1 v/v) in a separating funnel in three successive extraction cycles. A rotary evaporator (Buchi Rotavap RE IV) was used to concentrate the formed ethyl acetate extract at 40 °C and 170 mbar till complete dryness. The obtained crude extract was weighed 13.4 mg from 390 mL of broth was then dissolved in DMSO at 10 mg/mL and stored in a dark, moisture-free environment [21].

For all biological assays, the dried extract was freshly dissolved in dimethyl sulfoxide (DMSO) (Sigma-Aldrich-Germany) prior to use.

### Molecular identification of the marine bacterial isolate

#### DNA extraction and amplification by Polymerase Chain Reaction (PCR)

The isolate underwent molecular identification through 16 S rRNA gene sequencing [22]. The DNA of marine bacterial isolate was extracted using Genomic DNA extraction kit of the manufacturer, GeneJET. The sample’s DNA purity and concentration were assessed using a NanoDrop™ spectrophotometer (Thermo Fisher Scientific, USA), and DNA integrity was confirmed by 1% agarose gel electrophoresis [23].

The isolated DNA was used as a template to amplify the 16 S rRNA gene fragment by PCR utilizing universal primers 27 F (5′-AGAGTTTGATCCTGGCTCAG-3′) and 1492R (5′-TACGGYTACCTTGTTACGACTT-3′) in 50 µL reaction mixture [24]. The thermal conditions for PCR were as follows: an initial denaturation at 95 °C for 5 min, thirty cycles of 94 °C for 1 min, 55 °C for 1 min and 72 °C for 1 min, and concluded with a final extension at 72 °C for 5 min [17].

The PCR product (~ 1500 bp) was visualized by electrophoresis in 1% prepared agarose gel. This gel was prepared with Tris-borate-EDTA buffer at pH 8.3, supplemented with ethidium bromide (0.5 µg/ml). The ethidium bromide-stained amplicons were then viewed under an ultraviolet transilluminator.

#### DNa sequencing and internal transcribed spacer sequence analysis

Final PCR products were quantified via spectrophotometry and purified using micro spin filters. According to the manufacturer’s guidelines, the purified PCR products underwent Sanger sequencing with the ABI PRISM^®^ 3100XL Genetic Analyzer (Applied Biosystems, California, United States). To confirm the phylogenetic placement of the obtained 16 S rRNA gene sequence, similarity searches were performed against the NCBI GenBank database using BLASTn. Based on the BLAST results, adequate query coverage, sequence completeness and only type strains, the sequence of the isolated strain together with 10 closely related reference sequences were retrieved from GenBank. BLASTn algorithm against the NCBI database. Phylogenetic relationships were inferred using the Maximum Likelihood method [25] based on the Kimura 2-parameter model [26] All evolutionary analyses, including alignment and tree construction, were conducted in MEGA 11 [27]. Bootstrap values were calculated from 1,000 replicates to assess the reliability of the branching patterns.

### Antimicrobial activity assay (Agar Well Diffusion Assay)

The cup diffusion technique was used to evaluate the antimicrobial activity of the crude extract (10 mg/mL in DMSO) (CLSI, 2024). Test microorganisms were adjusted to a 0.5 McFarland turbidity standard (≈ 1.5 × 10⁸ CFU/mL) and uniformly spread onto Mueller–Hinton agar plates. Wells of 6 mm diameter were aseptically punched into the agar, and 50 µL of the crude extract solution was added to each well. Plates were incubated under aerobic conditions at 37 °C for 18–24 h, after which inhibition zone diameters were measured. Standard strains, *Escherichia* coli ATCC 25,922, *Pseudomonas aeruginosa* ATCC 27,853, *Staphylococcus aureus* ATCC 25,923 and *Candida albicans* ATCC 10,231 were used for the evaluation. The Microbiology and immunology Department, Faculty of Pharmacy, Ain Shams University, Cairo, Egypt provided these standard strains. The antifungal and antibacterial positive controls were ketoconazole (Sigma-Aldrich, Germany) and gentamicin (Sigma-Aldrich, Germany) at concentrations of 100 and 4 µg/mL, respectively, whereas negative control was DMSO to exclude solvent-related effects. Antimicrobial activity was proved according to the produced inhibition zones measured (mm).

### Cytotoxic effect (MTT Assay)

The used cell lines include WI-38 (Human Lung Fibroblast), A-549 (non-small cell lung carcinoma), HepG2 (hepatocellular carcinoma), HCT-116 (colorectal carcinoma), and MCF-7 (breast adenocarcinoma) were obtained from the regional center for mycology and biotechnology, Al Azhar University, Cairo, Egypt.

#### Cytotoxic effect against a normal cell line

The cytotoxic effect of the marine bacterial extract was figured out utilizing the MTT assay as reported by Manmuan et al. [28]. This technique was used to measure preliminary selectivity using the normal cell line WI-38. The cells were cultured in DMEM (Dulbecco Modified Eagle Medium) containing 1% LSPECTRA-glutamine, 10% heat-inactivated fetal bovine serum, HEPES buffer, and 50 µg/mL gentamycin.

#### Cytotoxic effect against tumor cell lines

Four different tumor cell lines, were utilized including A-549, HepG2, HCT-116, and MCF-7. On one side, the tumor cell lines were propagated in Roswell Park Memorial Institute medium (RPMI-1640) enriched with 10% inactivated fetal calf serum and 50 µg/mL gentamycin.

All cell lines were maintained at a temperature of 37°Cin an environment with 5% CO₂. They were sub-cultured two to three times each week to achieve an exponential phase. For assessment of cytotoxicity, the cells were seeded in 96 well microtiter plates and 1 × 10⁴ cells/well for tumor cell lines and a density of 1 × 10⁵ cells/well for WI-38 and were allowed to adhere for 24 h. The crude extract was checked for its cytotoxicity in 9–12 descending concentrations following the principle of the triplicated test. Negative controls comprised 0.1% DMSO or PBS, whereas 5-fluorouracil was the standard cytotoxic agent [29]. Following a duration of 48 h of exposure, each well received 100 µl of the medium and 10 µl of the MTT solution (12 mM; 5 mg/mL in PBS), which were then left for 4 h. The liquid was then drawn out, and each well was filled with 50 µL of DMSO to dissolve the formazan crystals.

Then, microtiter plates were incubated for an additional 10 min for the reaction, after which a microplate reader (SunRise, TECAN, Inc, USA) was used to measure at 590 nm. Cell viability was subsequently calculated as a percentage of the controls, and the inhibitory concentration (IC₅₀) values were nonlinearly adjusted with the use of GraphPad Prism software to determine the concentrations producing 50% non-toxicity. These IC₅₀ figures were used to measure the concentration of the extract that could cause the decomposition of cell viability to half, giving a comparative anticancer potency score for extract and cell lines.

### Anti-inflammatory activity

The in vitro cyclooxygenase-2 (COX-2) inhibition assay was used to evaluate the anti-inflammatory efficacy of the prepared extract, as described by Bin-Asal et al. [30] In that, the spectrophotometric detection of oxidized leuco-2,7-dichlorofluorescein (1-DCF) formed during the COX-2-mediated conversion of arachidonic acid to prostaglandins was the detection technique. In the beginning, leuco-DCF was formed by hydrolysis of 5 mg leuco-2,7-dichlorofluorescein diacetate in 50 µl of 1 M NaOH for ten minutes at room temperature. After that, 30 µl of HCl (1 M) was added for neutralization. For the experiment, the resultant 1-DCF was later combined with a 0.1 M Tris buffer (pH 8.0), allowing a suitable concentration, stability and pH of the buffer for enzymatic reaction. The COX-2 enzyme was also diluted in a 0.1 M Tris buffer (pH 8.0). Furthermore, it was pre-incubated for 5 min at room temperature with 20 µl of extract in the presence of hematin. Knowing that control is the substance needed for the enzyme to be activated. A prepared solution containing phenol (500 µM), 1-DCF (20 µM), and arachidonic acid (50 µM) were combined with the reaction mixture to start the enzymatic reaction and complete the final volume to 1 mL. A UV- visible spectrophotometer (Milton Roy, Spectronic 1201) was employed to monitor the reaction mixture at 502 nm for 1 min during that the absorbance of the 1-DCF was being oxidized. The UV-visible recordings were taken in triplicate to confirm the consistency and reproducibility of the inhibition data across concentrations and replicates.

To ensure that there is no chance of non-enzymatic oxidation and to make the findings of COX-2 inhibition unambiguous, the extract under investigation was subjected to an additional reaction without COX-2 enzyme being present at the same time the sample was analyzed. The operation was allowed to compensate for the existence of the intrinsic background of compounds, attending interaction with the extract and assay components. As a reference to an inhibitor of COX-2, celecoxib was utilized in the experiment conducted under the same assay conditions to secure the standard of the assay performance. The study tested microbial extract at 12 different concentrations to get a dose-response curve. According to the software of GraphPad Prism, IC₅₀ value was determined as the dose reducing 50% of the COX-2 enzyme activity.

### Antioxidant activity

The extract’s DPPH radical-scavenging activity was evaluated utilizing the DPPH assay described by Yen and Duh [31]. First, a fresh methanolic DPPH solution (0.004% w/v) was prepared. To evaluate the sample, 3 mL of the DPPH solution was mixed with a 40 µL aliquot of the extract (dissolved in methanol). A UV-Visible spectrophotometer (Milton Roy, Spectronic 1201) was used to measure the absorbance at 515 nm at one-minute intervals for 16 min. Each experiment was carried out in triplicate and the mean value for each concentration was determined. Under the same circumstances, ascorbic acid was utilized as a reference standard for comparison.

The tested extract’s free-radical scavenging ability was measured as DPPH radical percentage inhibition (PI) and determined as follows: PI = [(A_C_ - A_T_)/A_C_ x 100], where A_C_ represents the absorbance of the control and A_T_ denotes the absorbance of the treated sample after 16 min. To establish the dose-response curve, a two-fold serial dilution of the tested extract was prepared, ranging from 2 µg/mL to 1000 µg/mL.

The IC₅₀ value represents the concentration required to inhibit 50% of the DPPH radicals. The GraphPad Prism program was used to generate dose-response curves and determine the IC₅₀. The IC₅₀ value was therefore obtained graphically.

### Metabolite profiling of bacterial extract

The secondary metabolites in the crude extract were identified at the Proteomics and Metabolomics Unit at the Children’s Cancer Hospital (57357) in Cairo, Egypt. Liquid chromatography–electrospray ionization–tandem mass spectrometry (LC-ESI-MS/MS) was employed to analyze the crude extract after it was dissolved in a mixture of deionized water: methanol: acetonitrile (50: 25: 25). This analysis was performed with the ExionLC^™^ AD UPLC coupled to a TripleTOF 5600 + Time-of-Flight Tandem Mass Spectrometer (AB SCIEX) [32]. Attallah et al. [33] and Alqahtani et al. previously reported the adopted techniques [34].

### Statistical analysis

All the experiment was carried out in triplicate and the results were presented as means ± standard deviation (SD). To improve data clarity and reproducibility, descriptive statistics means and standard deviations), were calculated in Microsoft Excel 2016 (Microsoft Corp., Redmond, WA, USA). GraphPad Prism version 9.0 (GraphPad Software Inc., San Diego, CA, USA) was employed for graphical representations and statistical analyses utilizing one-way ANOVA and Tukey’s multiple comparison test. A P-value of less than 0.05 was considered statistically significant.

## Results

### Isolation of a marine bacterial isolate

Under the applied selective culture conditions, several bacterial colonies were recovered, but as the purpose of this work was designed as a preliminary bioactivity-oriented study, one isolate that showed consistent growth in liquid culture and detectable biological activity in initial screening assays was selected for further molecular identification and downstream analyses.

### Molecular identification via 16 S rRNA sequencing

According to the obtained results, the isolate belongs to the *Bacillus* genus within the Bacillaceae family and putatively assigned to *Heyndrickxia ginsengihumi* which coded MMM3 based on partial 16 S rRNA gene sequence analysis. The assignment of the bioactive isolate in the evolutionary tree of the Firmicutes phylum was supported both by a highly conserved 16 S rRNA gene region and detailed taxonomic analyses.

BLAST analysis of the 16 S rRNA sequence (GenBank accession: PX237387) revealed a sequence length of 1509 bp, with alignment spanning positions 4–1484, covering 98% of the sequence, and showing 99.93% nucleotide identity with *H. ginsengihumi* MMM3. The match consisted of 1480/1481 identical nucleotides, with an E- value of 0 and a bit score of 2728, confirming the robustness of this identification. Comparison was performed against a panel of reference strains, including their accession numbers, which provided further taxonomic support.

**Fig. 2:**
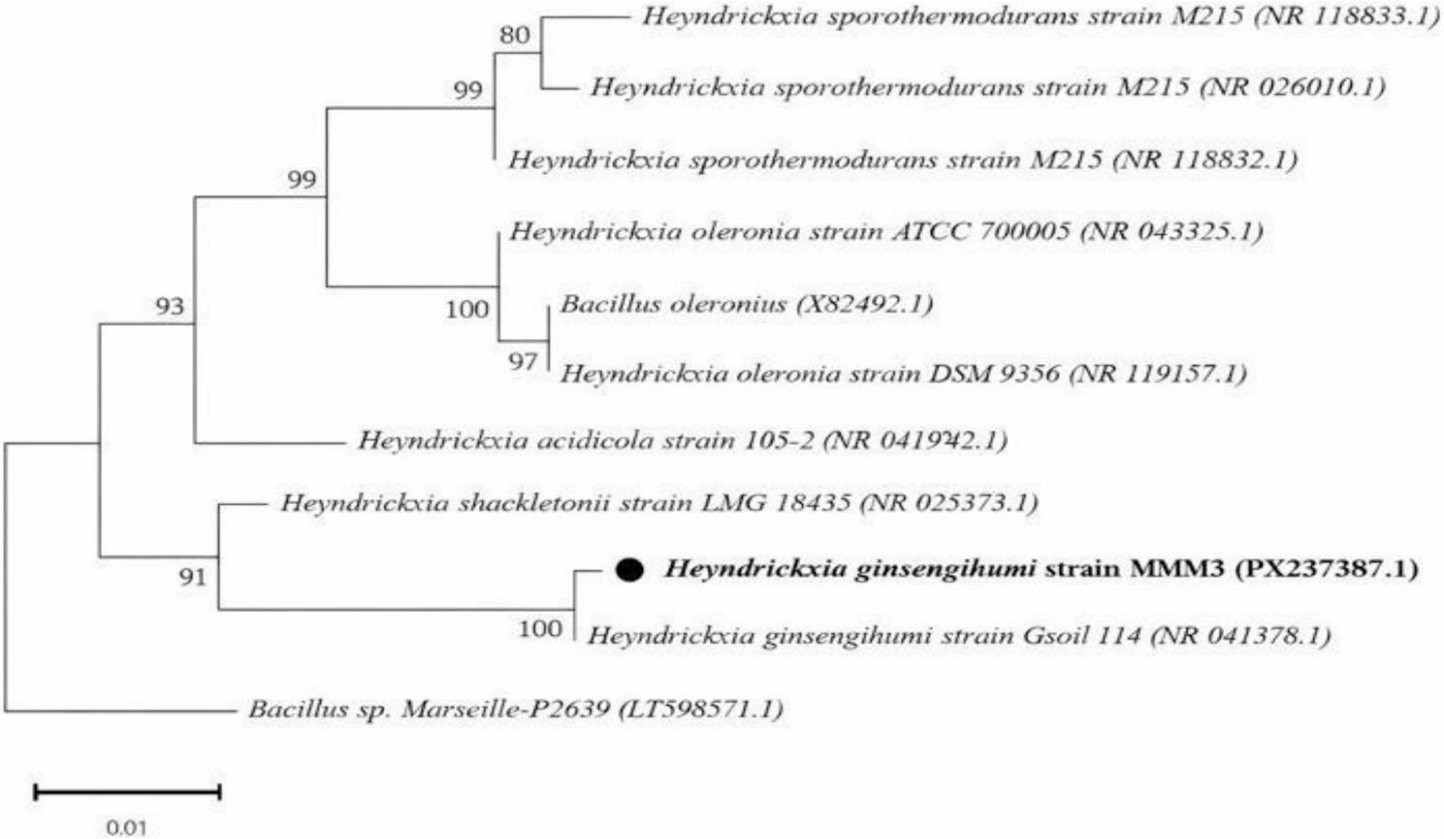
Maximum Likelihood phylogenetic tree based on 16 S rRNA gene sequences showing the taxonomic placement of the marine isolate *Heyndrickxia ginsengihumi* strain MMM3 (GenBank accession PX237387). The tree was constructed using the Kimura 2-parameter model with 1000 bootstrap replicates. Bootstrap support values (percentages) are shown at the corresponding nodes; branches with support below 50% collapsed. The scale bar represents 0.01 substitutions per nucleotide position. Analyses were performed using MEGA version 11. (alignment length 1499 bp)

Phylogenetic analysis based on the Maximum Likelihood method and the Kimura 2-parameter model placed isolate MMM3 within the Heyndrickxia clade, with strong bootstrap support (100%) for its clustering with the reference strain.Phylogenetic analysis based on the Maximum Likelihood method and the Kimura 2-parameter model placed isolate MMM3 within the Heyndrickxia clade, with strong bootstrap support (100%) for its clustering with the reference strain. The phylogenetic analysis of strain MMM3 is illustrated in Fig. 2.

### Antimicrobial activity

The antimicrobial efficacy of extract was evaluated against some selected reference microorganisms. The results obtained showed inhibitory activity only against *C. albicans* with inhibition zone 9 mm. The formed inhibition zone diameter of ketoconazole was 20 mm. Regarding the activity of the tested extract against the selected reference bacteria; no inhibition zone was observed.

### Cytotoxic activity

To assess the selective cytotoxicity of the tested extract, its cytotoxic activity was first evaluated against the normal human cell line WI-38. According to the results, the IC₅₀ of the crude extract was 172.75 ± 4.92 µg/mL. Regarding cytotoxicity of the crude extract against the investigated cancer cell lines HepG2, A-549, MCF-7, and HCT-116, the IC_50_ values were 54.67 ± 1.05, 60.19 ± 1.23, 73.57 ± 1.48, and 108.79 ± 2.08 µg/mL, respectively. The selectivity index (SI) of the crude extract was calculated by dividing the IC₅₀ value for normal cells (172.75 ± 4.92 µg/mL) by the IC₅₀ values for the tested cancer cell lines.

**Fig. 3:**
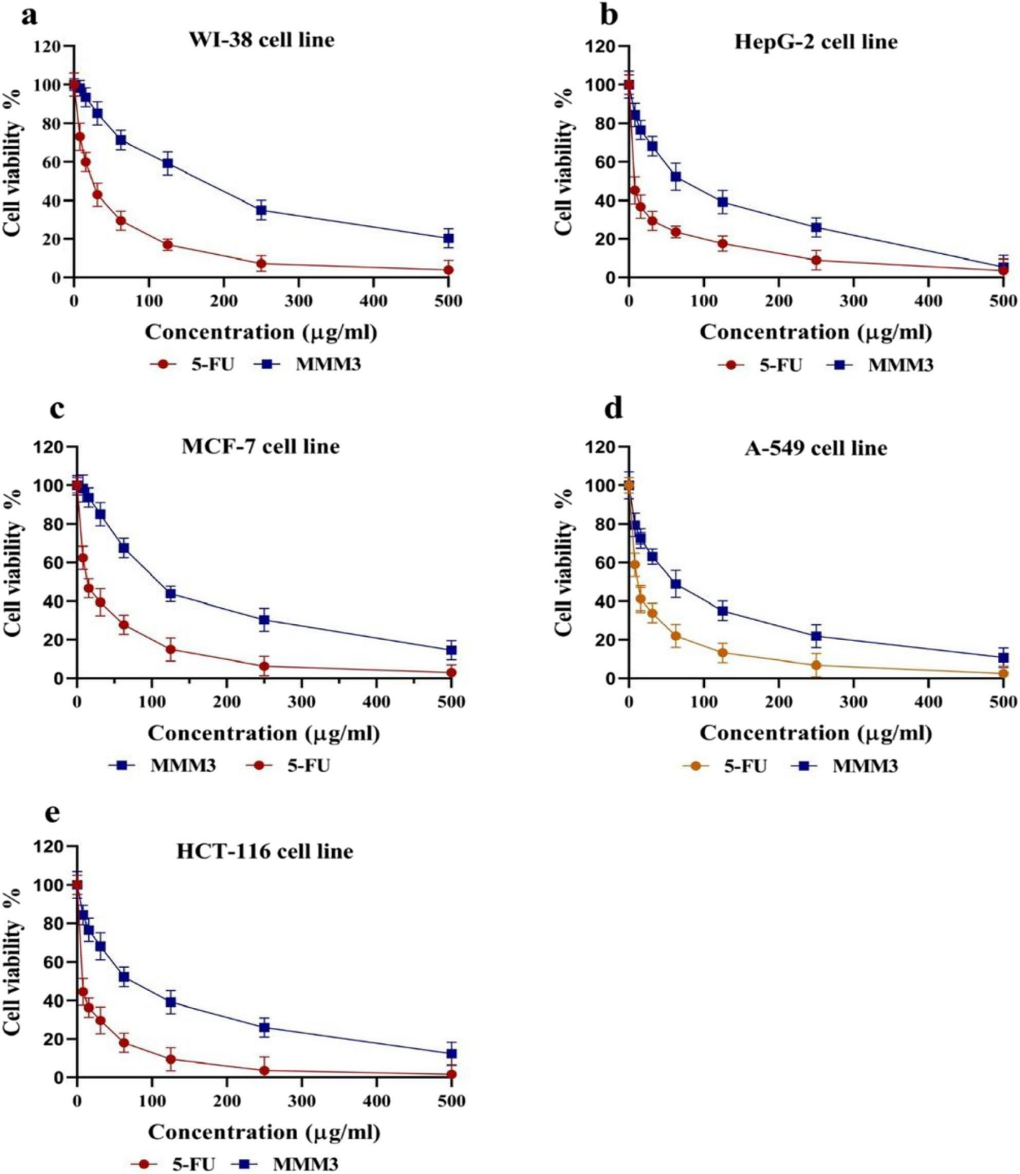
Dose-dependent effects of the crude extract (MMM3) compared with 5-fluorouracil (5-FU) on cell viability, as determined by the MTT assay. Cell viability (%) is shown as a function of concentration (µg/mL) for (**a)** WI-38 normal lung fibroblast cells, **b** HepG-2 hepatocellular carcinoma cells, **c **MCF-7 breast adenocarcinoma cells, **d** A-549 lung carcinoma cells, and (**e**) HCT-116 colorectal carcinoma cells. Data are expressed as mean ± SD of three independent experiments

The resulting SI values were 3.16, 2.87, 2.35, and 1.59 for HepG2, A-549, MCF-7, and HCT-116, respectively, indicating preferential cytotoxicity of the extract toward cancer cells compared with normal cells. The IC_50_ value of the positive control (5- fluorouracil) was 13.98 ± 0.75 µg/mL. **(**Fig. 3a-e**)** shows the dose-dependent cytotoxic effects of the crude extract and 5-FU on five cell lines.

### Anti-inflammatory activity (COX-2 Inhibition Assay)

**Fig. 4:**
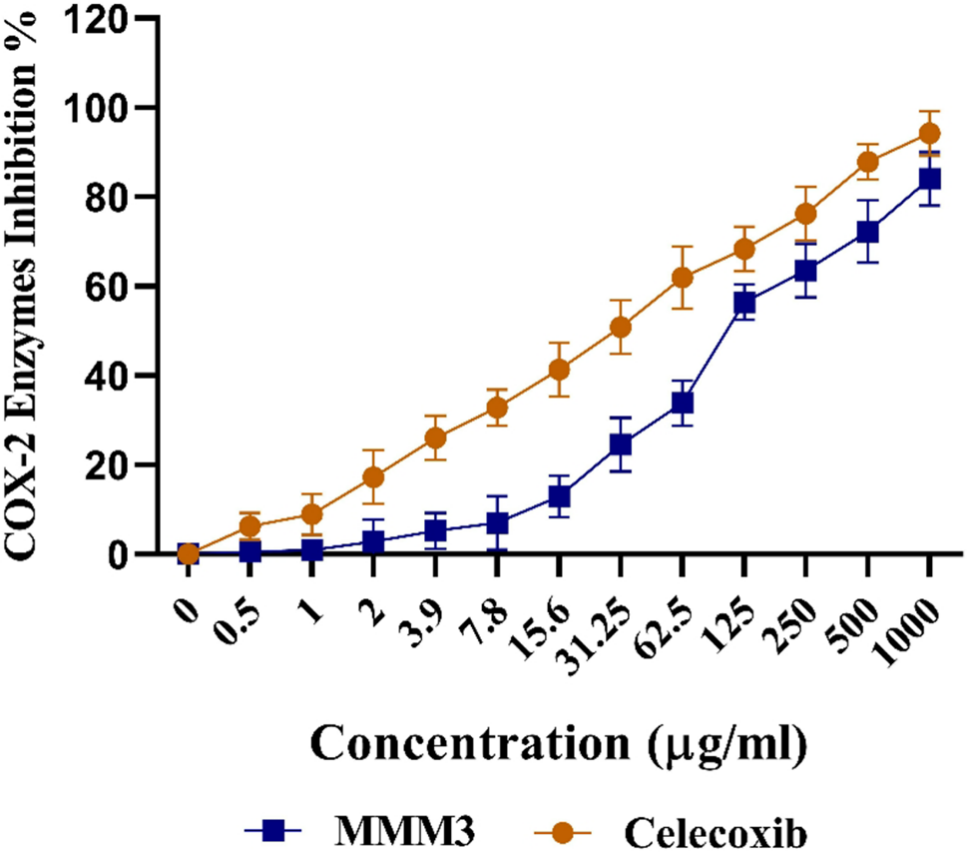
COX-2 inhibition effects of the crude extract and celecoxib. Data illustrates the anti-inflammatory potential of the *H. ginsengihumi* extract with an IC_50_​ of 107.07 ± 3.15 µg/mL compared to the reference inhibitor celecoxib

The COX-2 inhibition assay was used to assess the crude extract’s anti-inflammatory activity in vitro. The extract and positive control (Celecoxib) showed IC_50_ values of 107.07 ± 3.15 and 29.83 ± 1.51 µg/mL, respectively **(**Fig. 4**)**.

### Antioxidant activity (DPPH Assay Results)

**Fig. 5:**
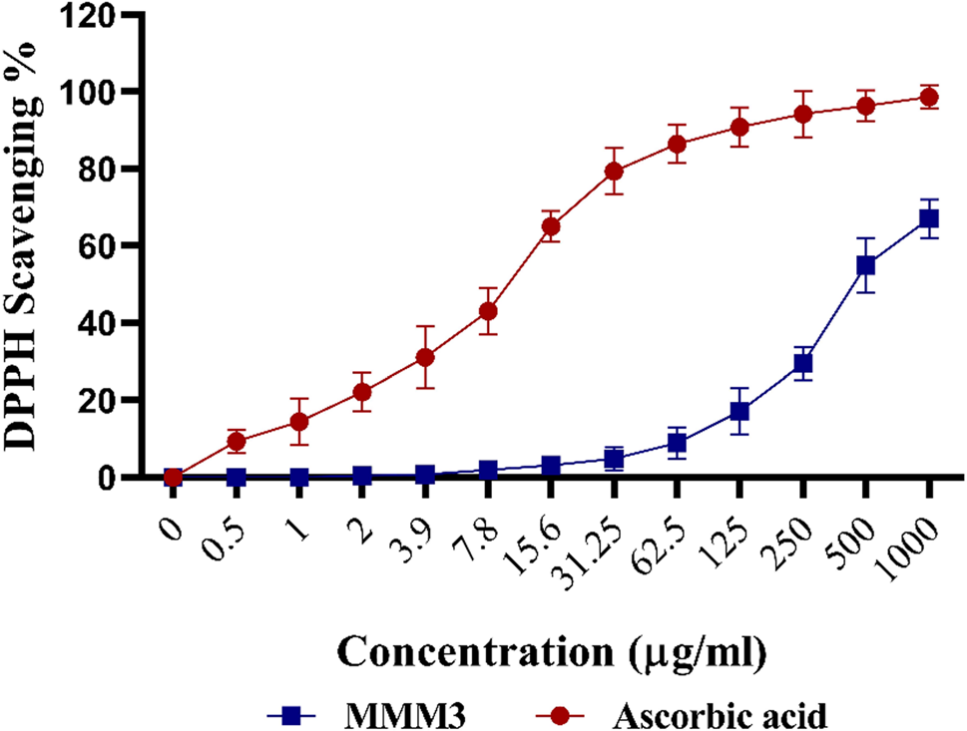
DPPH Scavenging Effect of the extract and ascorbic acid. The figure shows the free-radical scavenging percentage measured at various concentrations. The IC_50_​ of the crude extract was 451.75 ± 9.68 µg/mL relative to the ascorbic acid control

In the DPPH experiment, the radical scavenging percentage was measured using different concentrations of crude extract and ascorbic acid as a positive control. The results revealed that the IC_50_ of the crude extract was 451.75 ± 9.68 µg/mL. Regarding the ascorbic acid, the IC_50_ was 10.21 ± 0.77 µg/mL **(**Fig. 5**).**

### 3Metabolite Profiling of the Bacterial Extract

#### LC-ESI-MS/MS analysis of the *Heyndrickxia. ginsengihumi* bacterial extract

In this study, 26 compounds were tentatively identified from the bacterial extract using data-independent acquisition for mass spectral deconvolution with MS-DIAL in LC-ESI-MS/MS analysis (both positive and negative ESI modes). These compounds included flavonoids, fatty acids, sugar acids, amino acids, indole derivatives, diterpenoids, and dipeptides as shown in **(**Table 1**)**.

**Table 1:** Phytochemical profiling of *H. ginsengihumi* bacterial extract by LC-MS/MS analysis (negative and positive mode ESI)

Peak NO	Identification	Error ppm	*R*.T. (min.)	m/z	Adduct Ion	Ontology	Formula	MS/MS
1	N, N-Dimethylglycine	8.8	1.050	104.10	[M + H]^+^	alpha amino acids	C_4_H_9_NO_2_	56.04, 60.08, 104.10
2	L-Glutamic acid	-4.25	1.217	146.04	[M + H]^−^	Glutamic acid derivatives	C_5_H_9_NO_4_	74.02, 102.05, 128.03, 146.04
3	Glycyl-L-proline	4.45	1.334	173.09	[M + H]^+^	Dipeptides	C_7_H_12_N_2_O_3_	70.06, 116.07, 173.08
4	L-leucine	3.54	1.638	130.08	[M + H]^−^	Leucine derivatives	C_6_H_13_NO_2_	61.98, 84.07, 130.08
5	3-formylindole	-0.07	5.415	144.04	[M + H]^−^	Indoles	C_9_H_7_NO	65.99, 116.04, 144.04
6	[2-(4-hydroxyphenyl) ethyl] acetamide	1.67	6.203	180.10	[M + H]^+^	1-hydroxy-2-unsubstituted benzenoids	C_10_H_13_NO_2_	52.03, 65.03, 77.03, 93.03, 102.04, 180.10
7	L- (+)-tartrate	-9.58	7.328	149.02	[M + H]^−^	Sugar acids derivatives	C_4_H_6_O_6_	77.03, 105.03, 149.02
8	2-Hydroxyphenylacetic acid	-1.52	7.365	151.04	[M + H]^−^	2(hydroxyphenyl) acetic acids	C_8_H_8_O_3_	63.00, 106.04, 151.03
9	L-Threonic acid hemicalcium salt	-8.94	7.683	135.04	[M + H]^−^	Sugar acid derivatives	C_8_H_16_ CaO_11_	51.02, 65.03, 70.00, 92.02, 108.02, 135.04
10	*β*-indoleacetic acid	0.29	8.025	174.05	[M + H]^−^	Indole-3-acetic acid derivatives	C_10_H_9_NO_2_	65.99, 76.96, 86.97, 174.05
11	Alpha-Methyl-DL-serine	9.24	8.427	118.02	[M + H]^−^	Alpha amino acids	C_4_H_9_NO_3_	50.00, 90.03, 118.02
12	Naringenin	4.18	8.927	271.05	[M + H]^−^	Flavanones	C_15_H_12_O_5_	86.97, 110.97, 114.96, 152.98, 193.01, 221.01, 243.05, 271.04
13	N-(2-phenylethyl) acetamide	-0.06	9.004	164.10	[M + H]^+^	N-acetyl-2-arylethylamines	C_10_H_13_NO	51.02, 60.04, 77.03, 102.04, 105.07, 164.10
14	4-Hydroxy-3-methoxymandelate	8.05	9.376	197.02	[M + H]^−^	Methoxyphenols	C_9_H_10_O_5_	95.01, 121.02, 195.01, 197.02
15	Sebacate	0.70	10.164	201.11	[M + H]^−^	Medium-chain fatty acids	C_10_H_18_O_4_	57.03, 137.09, 183.10, 201.11
16	Quercetin	0.23	10.367	301.03	[M + H]^−^	Flavonols	C_15_H_10_O_7_	121.02, 151.00, 178.99, 232.92, 301.03
17	N-(9-oxodecyl) acetamide	0.75	10.977	214.18	[M + H]^+^	Acetamides	C_12_H_23_NO_2_	71.04, 137.13, 154.16, 172.17, 214.12
18	3,4-dihydroxy mandelate	7.63	11.048	183.00	[M + H]^−^	Catechols	C_8_H_8_O_5_	79.01, 95.01, 123.00, 153.00, 183.00
19	2’-deoxyuridine	9.33	13.273	227.02	[M + H]^−^	Pyrimidine 2’-deoxy ribonucleosides	C_9_H_12_N_2_O_5_	70.99, 83.99, 114.98, 158.97, 227.02
20	*β-*D-Glucopyranoside, phenylmethyl 6-*O*-[(2R,3R,4R)-tetrahydro-3,4-dihydroxy-4-(hydroxymethyl)-2-furanyl]-	11.1	14.134	425.13	[M + Na]^+^	*O*-glycosyl compounds	C_18_H_26_O_10_	90.97, 220.93, 360.85, 425.33
21	2-methoxy-4-pentadecyl benzoic acid	7.55	17.341	363.25	[M + H]^+^	*O*-methoxy benzoic acid derivatives	C_23_H_38_O_3_	90.97, 158.96, 226.95, 363.25
22	1-Myristoyl-sn-glycerol 3-phosphate	-7.64	21.304	381.23	[M + H]^−^	1-acylglycerol-3-phosphates	C_17_H_35_O_7_P	79.95, 96.95, 122.97, 379.99, 380.01
23	Palmitoleic acid	-1.97	21.81	253.21	[M + H]^−^	Long-chain fatty acids	C_16_H_30_O_2_	116.92, 252.39
24	Linoleic acid	-2.72	22.242	279.23	[M + H]^−^	Linoleic acid derivatives	C_18_H_32_O_2_	232.92, 261.22, 278.33
25	1-Naphthalenepentanol, decahydro-2-hydroxy-gamma,2,5,5,8a-pentamethyl-, alpha-acetate	-5.77	23.697	375.32	[M + Na]^+^	Diterpenoids	C_22_H_40_O_3_	374.12, 375.32
26	2-acetoxy-4-pentadecyl benzoic acid	3.97	25.75	413.26	[M + Na]^+^	Acyl salicylic acids	C_24_H_38_O_4_	57.07, 189.01, 301.14, 342.16, 411.95, 412.74

*RT* retention time, *m*/*z*: mass-to-charge ratio; ppm: 10^− 6^

**Fig. 6:**
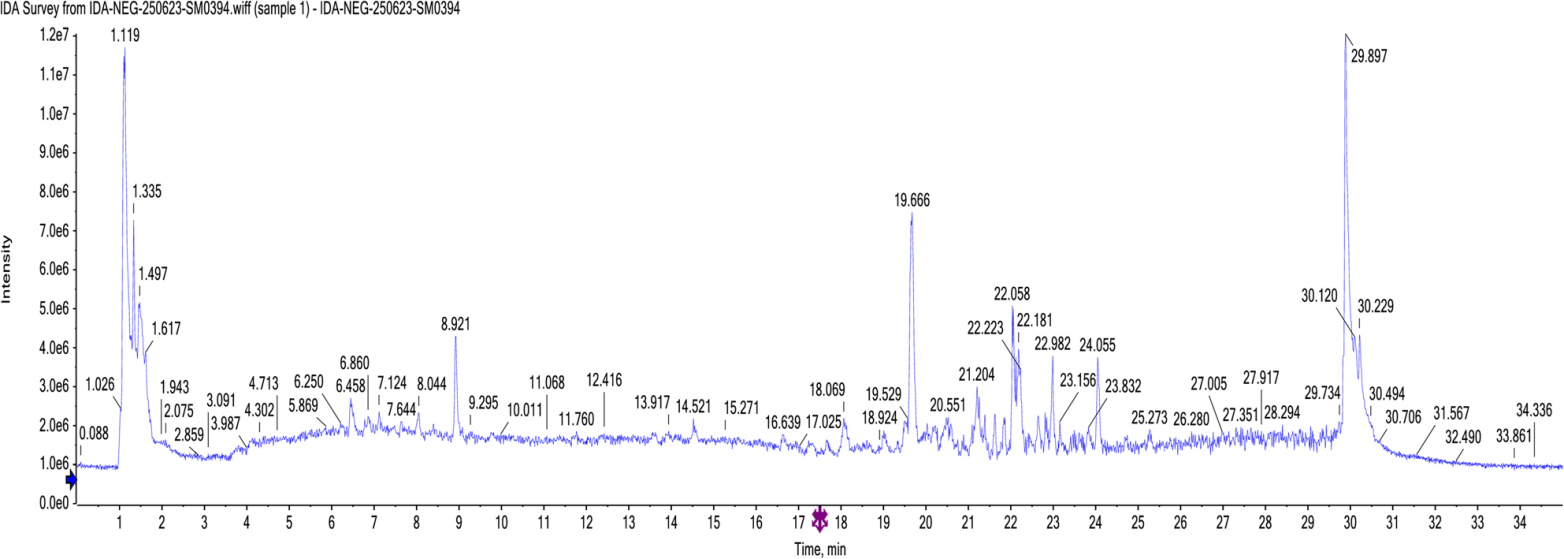
The Total Ion Chromatogram (TIC) of the tested extract of (negative mode)

**Fig. 7:**
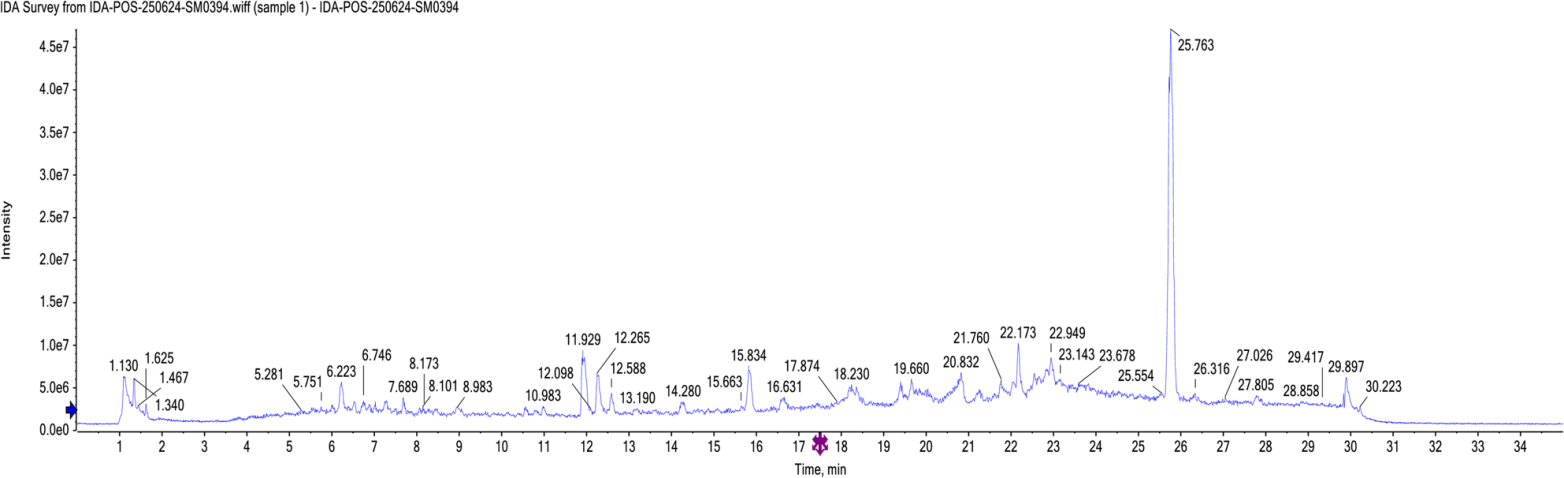
The Total Ion Chromatogram (TIC) of the tested extract of (positive mode)

**Fig. 8:**
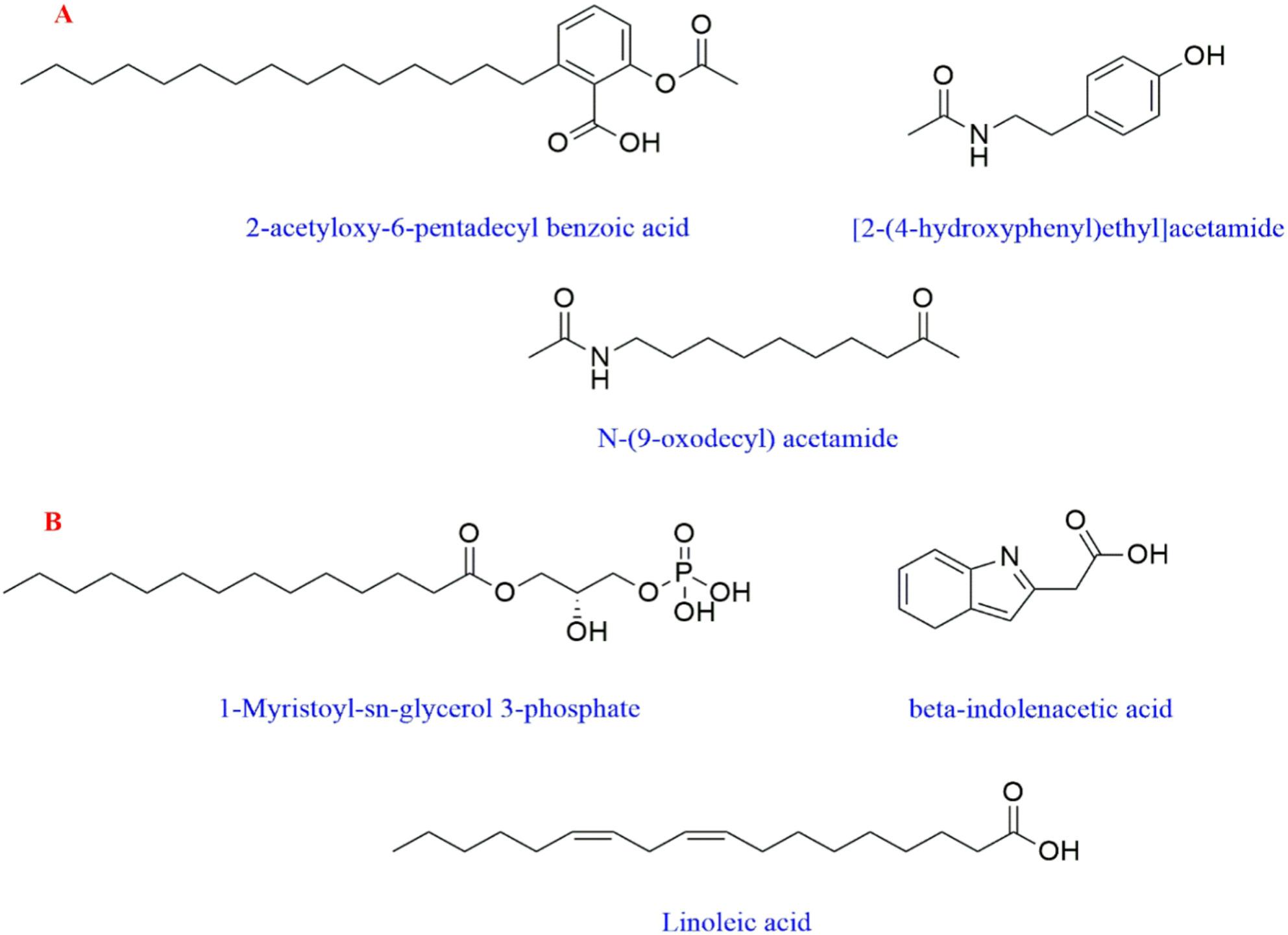
The chemical structures of the most prevalent compounds. Structural representations of major metabolites found in (**A**) Positive and (**B**) negative mode ESI

The Total Ion Chromatograms (TIC) of the *H. ginsengihumi* bacterial extract (negative and positive modes) are presented in (Figs. 6 and 7). In addition, the major compounds structures are presented in (Fig. 8).

l;

l;

## Discussion

The use of well-established methods and authorized protocols was implemented to ensure the precision and reproducibility of the findings. Thorough research on tunicates was centered around its importance in the ecosystem as a filter-feeding organism and its well-documented collaboration with marine associated bacteria that is likely in the synthesis of secondary metabolites [35]. Culture-based techniques are still used as standard methods to isolate bacterial strains from various environments. A marine bacterial isolate MMM3 was recovered from *Phallusia nigra* using R2A agar medium that was considered as one of the best media for isolating the marine bacteria [36]. Taxonomic identification of the isolate was based solely on 16 S rRNA gene sequencing and phylogenetic analysis. While this approach allowed preliminary identification as *Heyndrickxia ginsengihumi*, future work incorporating genome-based or multilocus analyses are planned to provide more definitive species-level confirmation.

The antimicrobial activity of different marine organisms was documented in earlier studies. The marine bacterial isolate *Bacillus licheniformis* exhibited antifungal activities. It was particularly active against a variety of fungi including *Aspergillus niger*, *Candida albicans*, and *Rhizoctonia solani* [37, 38]. In addition, Elfeky et al. [17] reported the antifungal activity of crude extract from a marine bacterial isolate coded HSP6N. The observed inhibition zone diameter against *Candida albicans* was 12 mm. The results of the biological activity of the marine isolate crude extract, in the present study, showed antifungal activity (9 mm inhibition zone) against *Candida albicans ATCC* 10231 but no antibacterial activity was observed. While the absence of antibacterial activity could theoretically be influenced by the diffusion kinetics of lipophilic metabolites or sub-threshold titers, the successful detection of a 9 mm antifungal zone validates the methodological rigor and extract potency. These results suggest that the secretome of *H. ginsengihumi* MMM3 is specifically characterized by antifungal properties under the utilized fermentation parameters, rather than broad-spectrum antibacterial activity [39] However, previous studies observed antibacterial activity induced by marine isolates. A marine *Streptomyces* isolate isolated from marine sponges of South Australia showed antibacerial activity against *Staphylococcus aureus* [40]. The marine isolate coded RAD3 demonstrated antibacterial activity against some Gram-positive as well as some Gram-negative bacterial strains [41]. The crude extract also demonstrated IC₅₀ values ranged from 54 ± 1.05–108.79 ± 2.08 µg/mL against cancer cell lines. These values indicated the concentration at which the extract achieves a 50% reduction in cell viability. Additionally, the IC₅₀ value against WI-38 (a normal cell line) was 172.75 ± 4.92 µg/ml indicating selective cytotoxic potential. The measured IC50​ values for the *H. ginsengihumi* MMM3 crude extract are considered moderate when compared to the purified drug 5-fluorouracil. However, within the scope of marine microbial bioprospecting, these values represent a significant biological signal. This level of activity in a crude state suggests that the active constituents are potent enough to manifest inhibitory effects despite being part of a complex matrix, warranting future bio-guided fractionation to isolate the pure bioactive compounds [42, 43]. 

Previous study showed IC₅₀ values of the crude extract of a marine *Streptomyces* isolate was ranging from 31.9 to over 100 µg/ml against different cancer cell lines [44]. The crude pigment extract obtained from marine *Pseudomonas aeruginosa* P1.S9 showed cytotoxic activity with IC₅₀ values 265.47 and 171.98 µg/ml against MCF-12 A and MCF-7 cell lines, respectively [45].

Regarding the anti-inflammatory activity, the crude extract demonstrated in vitro inhibitory activity on cyclooxygenase-2 (COX-2) with IC₅₀ = 107 ± 3.15 µg/ml. Hölken et al. [46]. reported that the marine-derived diterpene glycosides pseudopterosins A–D exhibit significant anti-inflammatory activity. Streptoglycerides E-H, extracted from the marine-derived bacterium *Streptomyces specialis*, showed notable effectiveness in reducing inflammation. These bioactive substances possessed anti-inflammatory properties [47].

To evaluate the antioxidant activity, the DPPH scavenging assay was utilized, revealing that tested extract showed IC₅₀ value of 451.75 ± 9.68 µg/mL which was in consonance with findings of Belhadj et al. [48]. and Ferdous et al. [49]. In their research, the ethyl acetate extracts of invasive seaweed *Rugulopteryx okamurae* and a marine microalgae *Tetraselmis* sp., respectively, showed potent antioxidant activity. In addition, Amasha [41] reported an antioxidant activity of marine bacterial isolate coded RAD3.

Along with the different experiments used for the biological activities’ evaluation of the crude extract of *Heyndrickxia ginsengihumi*, higher level of IC_50_ was reported when compared with that of the positive controls. This difference may be due to using crude extract of the tested isolate, while the positive controls were pure compounds.

Chemical analysis of the crude extract of *Heyndrickxia ginsengihumi* using LC-ESI-MS/MS was conducted to tentatively identify its bioactive components. Among the 17 secondary metabolites preliminary identified in the negative ESI mode, the major metabolites included 1-myristoyl-sn-glycerol 3-phosphate, *β*-indoleacetic acid, and linoleic acid. In the positive ESI mode, the main secondary metabolites among the nine metabolites detected were 2-acetoxy-4-pentadecylbenzoic acid, [2-(4-hydroxyphenyl)ethyl]acetamide, and N-(9-oxodecyl) acetamide [32]. The presence of linoleic acid and phenolic derivatives provides a plausible chemical basis for the recorded COX-2 inhibition (IC50​ 107.07 µg/mL) and antioxidant activity (IC50​ 451.75 µg/mL) as reported by Alam et al. [50]. Another secondary metabolite, 1-myristoyl-sn-glycerol-3-phosphate may have anticancer activity through a lipidation-enhanced mechanism. The identification of myristoyl glucose-3-phosphate in the present research may explain the observed anticancer activity through an enhanced lipidation-mechanism. While glucose-3-phosphate itself is a common metabolic intermediate with no direct cytotoxicity, the conjugation of a myristoyl group may alter its physicochemical properties. This modification (myristylation process) suggested to increase the compound’s lipophilicity, enhancing interaction with the plasma membrane and facilitating cellular uptake. Once internalized, the myristoyl group may localize to mitochondria or other membrane-rich organelles, leading to mitochondrial dysfunction and triggering apoptosis. This mechanism aligns with the findings of Li et al. [51]., who observed an increase of antitumor activity in myristoyl-CM4, where myristoylation improved membrane binding, enhanced cellular entry, and activated mitochondria-dependent apoptosis pathways. The anticancer activity may be related to the presence of another secondary metabolite, 2-acetoxy-4-pentadecylbenzoic acid, one of dietary benzoic acid derivatives. Similarly, benzoic acid derivatives had proven anticancer activity, as reported by Anantharaju et al. [52]. These annotations serve as high-priority leads for future bio-guided fractionation and quantitative validation, rather than experimentally confirmed mechanisms within the structural scope of the current work. While the biological activities were evaluated using crude extracts, it is important to note that these extracts represent complex mixtures of metabolites, which makes it difficult to attribute observed biological activity to specific metabolite. In addition, secondary metabolites may present at low concentrations leading to underestimated biological activity. Limited solubility or diffusion of certain compounds, especially in agar well diffusion assay and that masy mask antibacterial activity. This approach may explain preliminary insight into the bioactivity of the organism. Future work involving fractionation, purification, and characterization of specific metabolites will further clarify the mechanisms underlying the observed biological activities. The transition of *H. ginsengihumi* from terrestrial niches—exemplified by the type strain Gsoil 114—to the tissues of the marine tunicate *P. nigra* highlights a remarkable degree of ecological adaptation. While marine-associated *Bacillaceae* are well-documented producers of natural products, the isolation of strain MMM3 from the Red Sea provides a novel platform for exploring metabolic shifts driven by hypersaline environments. This investigation advances marine microbial research by bridging the gap between simple bioactivity screening and integrated ecological-chemical characterization. By documenting the multi-functional biological profile and the specialized metabolite suite of a marine-associated *H. ginsengihumi* strain, we provide evidence of how extreme marine environments like the Red Sea can drive metabolic adaptation and the production of bioactive constituents with therapeutic potential.

## Conclusion

A marine bacterial strain identified as *Heyndrickxia. ginsengihumi*, was isolated from the Red Sea coast. Its crude extract exhibited promising multiple variable biological activities including antifungal, anti-inflammatory, cytotoxic and antioxidant activities. Additionally, the crude contains different metabolites including fatty acids, sugar acids, amino acids, flavonoids, indole derivatives, diterpenoids, and dipeptides. Further study will be done to isolate these metabolites in purified forms and evaluate their biological activity separately.

## Data Availability

Identification of the bacterial strain was conducted through 16 S rRNA gene sequencing as *Heyndrickxia ginsengihumi* and was deposited in (NCBI) GenBank database under the accession code PX237387. The published manuscript contains full descriptions of all of the other data that was gathered and analyzed throughout the present investigation.

## Abbreviations

A_C_: Absorbance of the control
A_T_: Absorbance of the test
COX-2: Cyclooxygenase-2
DMEM: Dulbecco Modified Eagle Medium
DMSO: Dimethyl sulfoxide
5-FU: 5-Fluorouracil
1-DCF: Leuco-2,7-dichlorofluorescein
DPPH: 2,2-Diphenyl-1-picrylhydrazyl
IC_50_: Inhibitory Concentration 50%
LC-ESI-MS/MS: Liquid Chromatography–Electrospray Ionization–Tandem Mass Spectrometry
MTT: 3-(4,5-dimethylthiazol-2-yl)-2,5-diphenyltetrazolium bromide
NSW: Natural seawater
PCR: Polymerase Chain Reaction
PI: Percentage of inhibition
RPMI: Roswell Park Memorial Institute
RT: Retention time
TIC: Total ion chromatogram
